# Optical properties and bandgap evolution of ALD HfSiO_x_ films

**DOI:** 10.1186/s11671-014-0724-z

**Published:** 2015-02-05

**Authors:** Wen Yang, Michael Fronk, Yang Geng, Lin Chen, Qing-Qing Sun, Ovidiu D Gordan, Peng zhou, Dietrich RT Zahn, David Wei Zhang

**Affiliations:** 1 Institute of Advanced Nanodevices, School of Microelectronics, Fudan University, No. 220 Handan Road, Shanghai, 200433 China; 2 Semiconductor Physics, Technische Universität Chemnitz, Reichenhainer Straße 70, Chemnitz, D-09107 Germany

**Keywords:** HfSiO_x_, VUV, Bandgap

## Abstract

Hafnium silicate films with pure HfO_2_ and SiO_2_ samples as references were fabricated by atomic layer deposition (ALD) in this work. The optical properties of the films as a function of the film composition were measured by vacuum ultraviolet (VUV) ellipsometer in the energy range of 0.6 to 8.5 eV, and they were investigated systematically based on the Gaussian dispersion model. Experimental results show that optical constants and bandgap of the hafnium silicate films can be tuned by the film composition, and a nonlinear change behavior of bandgap with SiO_2_ fraction was observed. This phenomenon mainly originates from the intermixture of *d*-state electrons in HfO_2_ and Si-O antibonding states in SiO_2_.

## Background

With the downscaling of CMOS devices, high-*k* materials are required to replace SiO_2_ as gate dielectrics in order to decrease the direct tunneling leakage current and, at the same time, maintain the gate capacitance at a certain value [1-7]. Among the potential candidates, hafnium silicate was chosen as the first generation of high-*k* dielectrics for its high dielectric constant and excellent thermal stability [8,9]. Compared to other deposition methods used for hafnium silicate film fabrication, atomic layer deposition (ALD) has the advantages of precise film thickness and stoichiometry control, which are of great significance to optimize the material especially for the shrinking devices [10-15].

Since accurate determination of the optical properties is an essential prerequisite for device simulations and gives the opportunity to improve material preparation, we have applied vacuum ultraviolet (VUV) spectroscopic ellipsometry to investigate the optical characteristics of a set of hafnium silicate films in this work. It will also gain us an insight into the effect of film composition on the electrical performance and chemical states of hafnium silicate dielectric films.

## Methods

The targeted HfSiO_x_ thin films were deposited on lightly doped p-type Si (100) substrates by a BENEQ TFS-200 ALD system (BENEQ Oy, Espoo, Finland) at 200°C. The Si wafers were cleaned via RCA cleaning process at first, then prior to film growth, they were cleaned again in a diluted HF solution (50:1) to remove the native oxide and passivate the silicon dangling bonds followed by a deionized water rinsing and drying in N_2_. During deposition process, precursors for Hf, Si, and O were TEMAH, TDMAS, and O_2_ plasma respectively. TEMAH was kept at 80°C in a stainless bottle, and TDMAS was kept at room temperature. The O_2_ plasma was activated at the power of 100 W. Typical pulsing sequences during the ALD process are 1/3/2/2 s (TEMAH/Ar purge/O_2_ plasma/Ar purge) and 2/2/2/2 s (TDMAS/Ar purge/O_2_ plasma/Ar purge) for the growth of HfO_2_ and SiO_2_ films, respectively. For the HfSiO_x_ films, SiO_2_ percentage was controlled by deposition cycle ration of HfO_2_: SiO_2_. Pure HfO_2_, SiO_2_, and five groups of HfSiO_x_ samples with different atomic compositions were prepared.

For optical characterization, each sample was measured using a Woollam variable-angle vacuum ultraviolet spectroscopic ellipsometer (SE), and the data were analyzed with the software Complete EASE by J.A. Woollam. The measurements were taken at two angles of incidence, 67.5° and 75°, with a spectroscopic range of 0.6 to 8.5 eV. Then, to determine the optical properties of the target samples, such as layer thickness and optical constants, the model-based analysis were carried out. Complete EASE includes a wide range of built-in functions, such as Lorentz, Gaussian, Drude, Tauc-Lorentz, and Cody-Lorentz. These functions can be used to approach a wide variety of thin film, ranging from dielectrics and organics to semiconductors and metals. In this work, the Cauchy dispersion relation was adopted for the determination of the films thickness and the optical properties were analyzed with the Gaussian dispersion model.

## Results and discussion

The low energy range of each spectrum (0.6 to 4 eV) was used to determine the thickness of the sample by Cauchy dispersion relation, which is often adopted to describe the refractive index for transparent films in the visible spectral range. The Cauchy formula can be given by $n\left(\uplambda \right)=A+\frac{B}{\lambda^2}+\frac{C}{\lambda^4}$. The extinction coefficient *k* equals 0 at all used wavelengths. In this equation, the ‘*A*’ parameter relates to the approximate amplitude for the material index, while ‘*B*’ and ‘*C*’ parameters provide the shape and curvature of the index versus wavelength. Film thicknesses of each sample obtained from Cauchy dispersion relation are listed in Table 1 (in the table HfSiO_x_ films are denoted as (HfO_2_)_1-*x*_(SiO_2_)_*x*_, where *x* refers to different Si concentrations) together with the MSE values. ‘MSE’ is an acronym for mean squared error. It is the metric used to quantify the agreement between the experimental data and the parameterized optical model. ‘Perfect’ agreement would yield a MSE value equal to 0, and large deviations away from 0 will lead to erroneous extracted physical values (i.e., thickness and optical constants) [16,17]. From Table 1, it can be seen that for all samples, MSEs are very small values, and error bar of each sample is at least 2 magnitudes smaller than the thickness obtained, proving the correctness of extracted thickness.

**Table 1:** **Thickness of samples by Cauchy dispersion relation**

	**Thickness (nm)**	**MSE**
HfO_2_	9.80 ± 0.015	0.613
(HfO_2_)_0.95_(SiO_2_)_0.05_	12.00 ± 0.020	0.983
(HfO_2_)_0.8_(SiO_2_)_0.2_	10.39 ± 0.021	0.810
(HfO_2_)_0.6_(SiO_2_)_0.4_	9.36 ± 0.026	1.035
(HfO_2_)_0.4_(SiO_2_)_0.6_	8.80 ± 0.026	1.019
(HfO_2_)_0.2_(SiO_2_)_0.8_	11.36 ± 0.039	0.661
SiO_2_	11.13 ± 0.049	0.641

MSE = mean squared error.

To analyze the optical properties (behavior of refractive index and extinction coefficient), film thickness acquired above were fixed and Gaussian dispersion model was used with the fitted range expanded to 0.6 to 8.5 eV. The Gaussian oscillator features a Gaussian line shape for the imaginary part of the complex dielectric function *ε*_2_, with a Kramers-Kronig consistent line shape for the real part of the dielectric function *ε*_1_ [18]:

1 $$ {\upvarepsilon}_{n2}={A}_n{e}^{-\left(\left(E-{E}_n\right)/{B}_{rn}\right)}+{A}_n{e}^{-\left(\left(E+{E}_n\right)/{B}_{rn}\right)} $$

2 $$ {\varepsilon}_{n1}={\varepsilon}_1\left(\infty \right)+\frac{2}{\pi }P{\displaystyle \underset{E_g}{\overset{\infty }{\int }}}\frac{\zeta {\upvarepsilon}_{n2}\left(\zeta \right)d\zeta }{\zeta^2-{E}^2} $$

In the equations above, the *P* stands for the Cauchy principal part of the integral. The Gaussian oscillator model employs four setting parameters: the amplitude *A*, the broadening parameter *B*_r_, the center energy *E*_n_, and the non-dispersive term *ε*_1_ (∞). The two fitting parameters *B*_r_ and *E*_n_ are in units of energy while *A* and *ε*_1_ (∞) are dimensionless. *ε*_1_ (∞) represents the contribution of the optical transitions at higher energies and appears as an additional fitting parameter [19].

**Figure 1:**
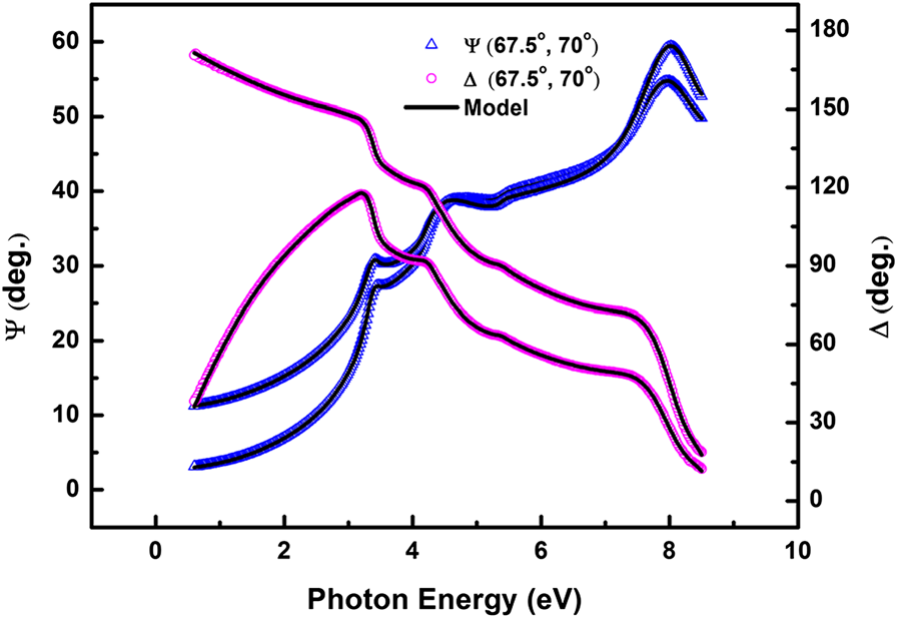
**Experimental and fitted spectroscopic ellipsometric data Ψ and Δ for HfSiO**_**x**_ **sample with 80% SiO**_**2.**_

As an example, the experimental SE data *Ψ* and Δ of HfSiO_x_ sample with 80% SiO_2_ are shown in Figure 1. For both incidence angles, fairly good agreement between the experimental and fitted spectra are clearly demonstrated, revealing that the Gaussian model works well and optical constants of the film can be exactly determined by the best fitting results. Data of other samples are not shown here due to space limitations, but fitted data for all samples match as well with the experimental data.

**Figure 2:**
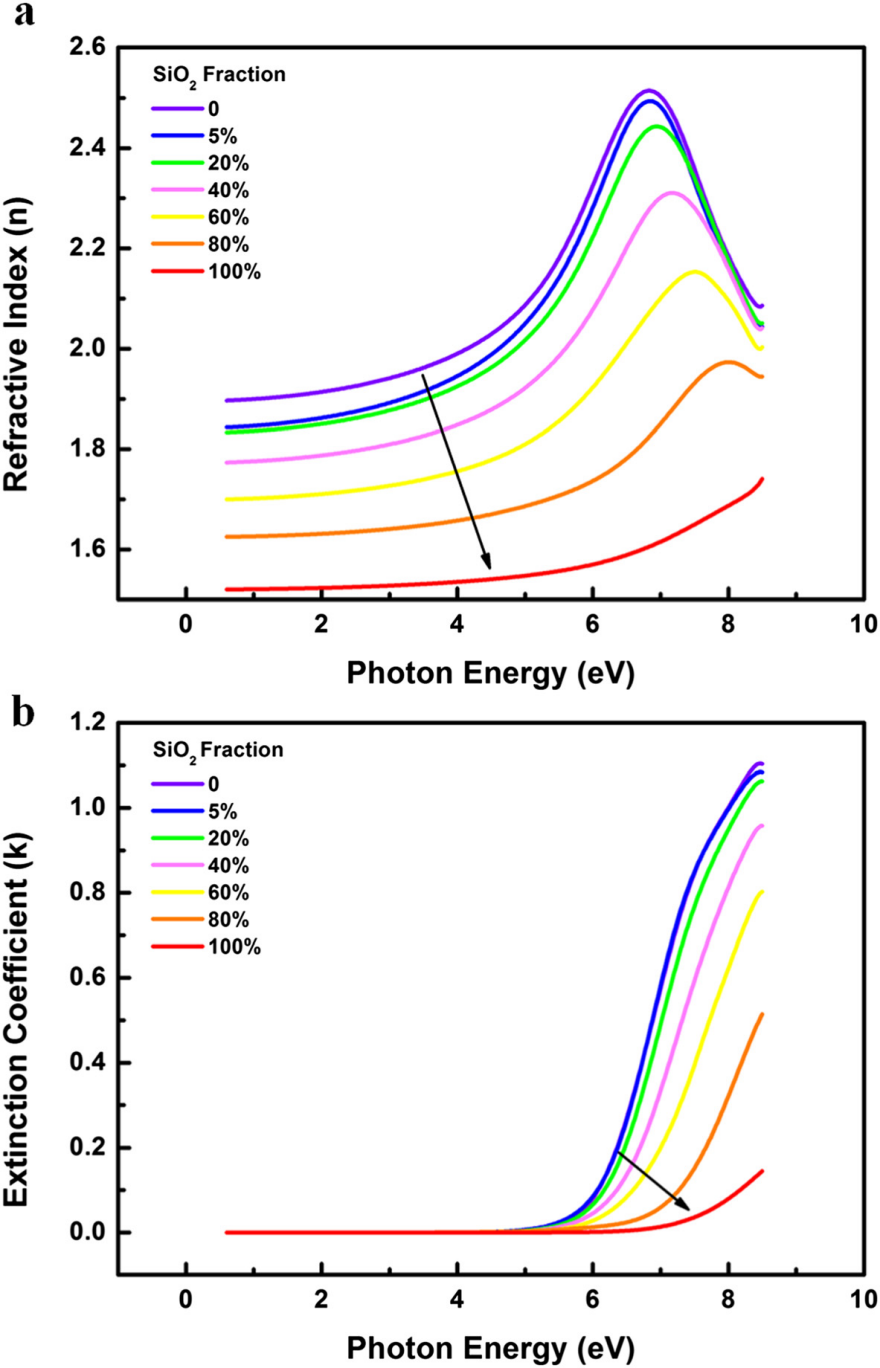
**Calculated** ***n*** **(a) and** ***k*** **(b) of the HfSiO**_**x**_ **films with different SiO**_**2**_ **incorporation content.**

Figure 2a shows the refractive index *n* as a function of photon energy for all samples as deduced from the analysis of the SE results. The *n* value for HfO_2_ measured at 550-nm (2.26 eV) wavelength is 1.84, which is similar with the previous report [20]. In addition, as shown in Figure 2a, the index of refraction decreases with the increase of Si concentration in the films. According to the Lorentz-Lorenz relation [21], the refractive index can be related to the evolution of packing density and polarization. Since Si-O bonds tend to be less polar than the corresponding Hf-O bond [22], the increase of Si concentration in the film would lead to a decrease of the film polarization, then lower polarizability results in the lowering of the refractive index.

The evolution of extinction coefficient *k* is shown in Figure 2b. For all samples, *k* saturates to zero in the visible region, suggesting the realization of high quality HfSiO_x_ films in terms of optical properties. An abrupt increase in the extinction coefficient for higher photon energy is due to the fundamental bandgap absorption in the films. Moreover, as can be seen in Figure 2b, there is also a mall band tail below the gap. This weak absorption is attributed to the Urbach tail which exists below the bandgap of amorphous materials and due to the disorder of the amorphous network [23,24]. Furthermore, with the increase of Si concentration, decrease in the magnitude of the exponential tail can be observed, and similar results were obtained by the Cody-Lorentz model and Tauc-Lorentz model [16]. According to J. Price et al., this phenomenon is attributed to Si atoms filling the O_2_ vacancies/defects in the HfO_2_ films. By filling these vacancies, there is less disorder and therefore, less intraband absorption.

**Figure 3:**
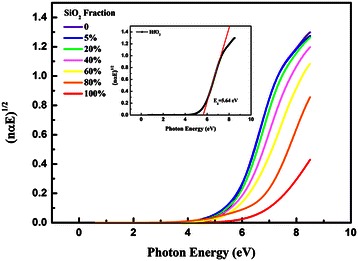
**Determination of the optical bandgap.**

Optical bandgap of the films were determined by plotting the empirical expression (*nαhν*)^1/2^ versus *hν*, as shown in Figure 3, where *n*, *α*, and *hν* stand for the index of refraction, the absorption coefficient, and the photon energy, respectively. The absorption coefficient *α* can be easily obtained by the equation *α* = 4π*k*/*λ*, where *λ* is the wavelength of the incidence light [25]. By extrapolating the straight near the band edge to zero, the crossing point with the *x*-axis is considered to be the optical bandgap of the film. To make it clear, bandgap determination of HfO_2_ is taken as an example and is shown in the inset of Figure 3. The extracted bandgap of pure HfO_2_ film is 5.64 eV, in good agreement with the previously reported values 5.25 to 5.8 eV for HfO_2_ [23,26,27].

**Figure 4:**
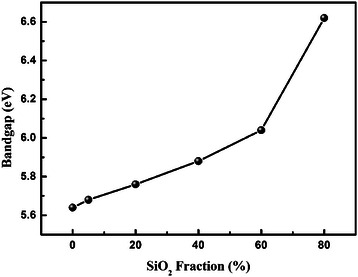
**Bandgap evolution of the HfSiO**_**x**_ **sample with different SiO**_**2**_ **incorporation content.**

Based on the method depicted above, the set of optical bandgaps acquired in Figure 3 is plotted in Figure 4 for details. As an exception, the bandgap of pure SiO_2_ is not shown in Figure 4, because although the fitted range is sufficient for most of the samples, it is not for pure SiO_2_ and the linear part of the curve in Figure 3 has not appeared yet. It is clearly demonstrated in Figure 4 that with increasing Si concentration, there is a monotonically increase in the bandgap of the films. This change mainly originates from the slight difference in electronic structure. And if we go into details, it can be found that *E*_g_ increases rapidly when SiO_2_ fraction is more than 60%. The similar nonlinear change of HfSiO_x_ bandgap with Si concentration was also observed by others [28,29]. It is known that the nonbonding O 2*p* states form the top of the SiO_2_ valence band and that the Si-O antibonding states form the bottom of its conduction band [17]. In the case of HfO_2_, the top of valence band are also composed of O 2*p* states but the bottom of conduction band states are mostly composed of Hf localized 5*d* state [28]. Similar to the explanation given by H. Kato et al. in the case of ZrSiO_x_ film [29], the rapid decrease in *E*_g_ with an increase in Hf concentration is considered to be attributed to the increase in the *d*-state electrons. When Si fraction is lower than 0.6, it seems that the bottom of the conduction band is almost formed by the Hf *d*-states, leading to the gradual decrease in *E*_g_.

## Conclusions

The optical properties of ALD hafnium silicate films, together with pure HfO_2_ and SiO_2_ films as references, were investigated systematically based on Gaussian dispersion model. According to the results, optical constants and bandgap of the hafnium silicate films can be tuned by the film composition, and a nonlinear change behavior of bandgap with SiO_2_ fraction was observed. This phenomenon mainly originates from the intermixture of *d*-state electrons in HfO_2_ and Si-O antibonding states in SiO_2_.
