# Erratum to: Optical properties and bandgap evolution of ALD HfSiO_x_films

**DOI:** 10.1186/s11671-015-1079-9

**Published:** 2015-09-29

**Authors:** Wen Yang, Michael Fronk, Yang Geng, Lin Chen, Qing-Qing Sun, Ovidiu D. Gordan, Peng Zhou, Dietrich R. T. Zahn, David Wei Zhang

**Affiliations:** Institute of Advanced Nanodevices, School of Microelectronics, Fudan University, No. 220 Handan Road, Shanghai, 200433 China; Semiconductor Physics, Technische Universität Chemnitz, Reichenhainer Straße 70, Chemnitz, D-09107 Germany

The authors of Nanoscale Research Letters 2015, 10:32 (DOI 10.1186/s11671-014-0724-z) [1] omitted to acknowledge that all ellipsometric data discussed in the article, including those displayed in Figure 1, were recorded in the laboratory of the Semiconductor Physics Group, Institut für Experimentelle Physik, Universitāt Leipzig, with the active involvement and under the guidance of Tammo Böntgen, Rüdiger Schmidt-Grund, and Marius Grundmann.
